# Predictors of Severe Outcomes in COVID-19: Evidence from Real-World Multicenter Retrospective Study (2020–2024)

**DOI:** 10.3390/jcm15031207

**Published:** 2026-02-03

**Authors:** Małgorzata Wajdowicz, Krystyna Dobrowolska, Kinga Brzdęk, Jakub Janczura, Dorota Zarębska-Michaluk, Łukasz Supronowicz, Piotr Rzymski, Magdalena Rogalska, Piotr Czupryna, Krzysztof Tomasiewicz, Marcin Hawro, Michał Brzdęk

**Affiliations:** 1 Clinical Department of Infectious Diseases with Hepatology Subdivision, Collegium Medicum University of Rzeszow, 35-959 Rzeszow, Poland; m.wajdowicz@gmail.com (M.W.); mhawro@mp.pl (M.H.); 2 Collegium Medicum, Jan Kochanowski University, 25-516 Kielce, Poland; krystyna.dobrowolska98@gmail.com (K.D.); kuba.janczura@gmail.com (J.J.); michal.brzdek@gmail.com (M.B.); 3 Department of Rheumatology and Connective Tissue Diseases, Nicolaus Copernicus Memorial Hospital, 93-513 Lodz, Poland; brzdekinga@gmail.com; 4 Department of Infectious Diseases and Allergology, Jan Kochanowski University, 25-516 Kielce, Poland; 5 Department of Infectious Diseases and Hepatology, Medical University of Bialystok, 15-540 Bialystok, Poland; l.supronowicz@wp.pl (Ł.S.); pmagdar@gmail.com (M.R.); 6 Department of Environmental Medicine, Poznań University of Medical Sciences, 60-806 Poznan, Poland; rzymskipiotr@gmail.com; 7 Department of Infectious Diseases and Neuroinfections, Medical University of Bialystok, 15-540 Bialystok, Poland; avalon-5@wp.pl; 8 Department of Infectious Diseases, Medical University of Lublin, 20-081 Lublin, Poland; tomaskdr@poczta.fm; 9 Department of Gastroenterology, Medical University of Lodz, 92-213 Lodz, Poland

**Keywords:** COVID-19, sex differences, clinical outcomes, mechanical ventilation, inflammatory markers

## Abstract

**Background/Objectives**: This study analyzed demographic, clinical, laboratory, and outcome data from patients hospitalized with Coronavirus disease 2019 in eastern Poland between March 2020 and December 2024. This study aimed to assess sex-related differences in clinical features, treatments, and outcomes, and to identify predictors of mortality and mechanical ventilation in hospitalized patients. **Methods**: A retrospective cohort of 2811 adults hospitalized across four infectious disease centers was examined. Data included demographics, comorbidities, symptoms, laboratory findings, treatments, and clinical outcomes. Multivariable logistic regression was performed to identify predictors of mortality and mechanical ventilation. **Results**: The cohort comprised 1398 females and 1413 males. Women were older (median 67.5 vs. 63 years, *p* < 0.0001) and had a higher burden of comorbidities, while men presented with more severe baseline respiratory status and higher inflammatory markers. Oxygen therapy was required more frequently in men (60% vs. 49.9%, *p* < 0.0001). Overall mortality was 8.9% and did not differ significantly by sex, although men aged 60–79 years had higher mortality than women (11.2% vs. 7.7%, *p* = 0.0422). Independent predictors of mortality (OR, 95%CI) included age ≥ 80 years (3.78, 2.66–5.39), procalcitonin > 1 ng/mL (OR 4.07, 2.54–6.52), interleukin-6 (IL-6) > 100 pg/mL (OR 2.24, 1.53–3.27), and oxygen therapy at admission (OR 9.41, 5.22–16.97). Predictors of mechanical ventilation were age ≥ 80 years (7.14, 1.75–33.33), procalcitonin > 1 ng/mL (OR 2.09, 1.2–3.63), IL-6 > 100 pg/mL (OR 2.3, 1.4–3.78), and CRP at admission (OR 1.82, 1.15–2.88). **Conclusions**: Sex-related disparities in clinical presentation, laboratory profiles, and treatment strategies were evident, but mortality differences were driven primarily by age and inflammatory burden rather than sex alone. Elevated procalcitonin, high IL-6, and early oxygen requirement emerged as robust predictors of poor outcomes.

## 1. Introduction

Coronavirus disease 2019 (COVID-19), caused by severe acute respiratory syndrome coronavirus 2 (SARS-CoV-2), remains a global health challenge more than five years after its initial emergence [1]. Although the acute impact of the pandemic has diminished compared with the 2020–2022 period, the World Health Organization (WHO) continued to classify COVID-19 as a condition posing a high global public health risk [2]. This assessment reflects widespread population immunity, acquired through vaccination and prior infection, as well as improved clinical management and sustained access to diagnostics [2].

As of 24 December 2024, the WHO reported more than 776.8 million confirmed cases of COVID-19 and over 7 million deaths worldwide. The true burden is likely much higher due to underreporting and limited testing capacity [1,2]. Approximately 80% of infections are asymptomatic or mild [1,3]; however, SARS-CoV-2 may still lead to severe outcomes such as pneumonia, respiratory failure, and multiorgan dysfunction. Although general population-level mortality has declined to approximately 1% in recent years, hospitalized patients remain at substantially higher risk [3,4,5,6,7,8]. Mortality further increases in the presence of COVID-19-related complications, particularly multi-organ failure and the need for advanced organ support. Reported mortality rates reach 66.1% in patients receiving renal replacement therapy, 58.0% among those treated with extracorporeal membrane oxygenation, and range from 39% to 70% in patients with COVID-19-associated acute respiratory distress syndrome (ARDS) [3,4,5,6,7,8].

The clinical course of infection is shaped by a combination of host, viral, and healthcare-related factors. Viral determinants are linked to the ongoing evolution and emergence of new variants. The WHO classifies variants into three categories: Variants of Concern (VOCs), Variants of Interest (VOIs), and Variants Under Monitoring (VUMs). To date, five VOCs have been identified: Alpha, Beta, Gamma, Delta, and Omicron. Although no SARS-CoV-2 variant currently meets the criteria for a VOC, Omicron and its sublineages dominate globally, with JN.1 and its descendants accounting for more than 99% of reported sequences by late 2024. These variants exhibit immune-escape properties but have not demonstrated increased intrinsic virulence compared with earlier Omicron strains [1].

Host-related characteristics remain central determinants of clinical outcome. Advanced age is consistently among the strongest predictors of severe disease and death, with risk rising sharply in individuals aged ≥60 years and particularly those aged over 80 [7,9,10]. The presence of comorbidities—including arterial hypertension, diabetes, cardiovascular and chronic respiratory disease, malignancy, and immunosuppression—further amplifies risk. Male sex has repeatedly been associated with a higher probability of severe disease and intensive care admission, potentially reflecting differences in immune response, hormonal regulation, angiotensin-converting enzyme 2 expression, and behavioral factors such as smoking prevalence [11,12]. However, the role of sex is not uniform; some studies report that excess male mortality diminishes after adjusting for age and comorbidity burden, while others emphasize sex-specific immune and inflammatory patterns influencing outcomes [11,13]. This inconsistency underscores the need for further population-specific analyses.

In addition to demographics and comorbidities, laboratory indicators of systemic inflammation, notably elevated C-reactive protein (CRP), interleukin-6 (IL-6), and procalcitonin (PCT), are strongly associated with disease progression and adverse outcomes [14]. Similarly, the requirement for oxygen therapy at hospital admission serves as a practical early marker of clinical deterioration and a predictor of mechanical ventilation and mortality [15,16]. In contrast, vaccination and prior infection provide protection, generally mitigating disease severity [17,18].

During the early phase, patient management relied largely on supportive care, as no therapies with proven clinical efficacy were available, which likely contributed to the high mortality observed among hospitalized patients with severe disease. As evidence from randomized clinical trials accumulated, the management of severe COVID-19 increasingly incorporated evidence-based therapeutic strategies. These included antiviral therapy in the early phase of infection, the use of monoclonal antibodies in selected high-risk patients, immunomodulatory treatment targeting hyperinflammation, systemic corticosteroids in patients requiring oxygen supplementation, and anticoagulation to reduce thromboembolic risk [19,20]. The gradual implementation of these strategies was associated with improved outcomes across successive pandemic waves, although healthcare system strain during peak periods continued to contribute to excess mortality [3,10].

Although global analyses provide valuable insights, they may obscure country-specific differences related to local epidemiology, healthcare infrastructure, and vaccination strategies. In Poland, as in many other European nations, the decline in case fatality has coincided with reduced testing and reporting, making hospital-based cohorts an essential source of reliable clinical data [1,21].

Therefore, this study aimed to compare the epidemiological characteristics, clinical presentation, laboratory parameters, treatment patterns, and outcomes of men and women hospitalized with COVID-19, and to conduct a multivariable analysis of predictors of mechanical ventilation and in-hospital mortality.

## 2. Materials and Methods

### 2.1. Study Population and Data Collection

We conducted a retrospective analysis using data from 2811 adult patients, hospitalized in four medical centers located in eastern Poland (Bialystok, Kielce, Lublin, and Lancut) between March 2020 and December 2024. The diagnosis of SARS-CoV-2 infection was confirmed by either a positive polymerase chain reaction or antigen test. Patient management and treatment were carried out in accordance with current national recommendations for COVID-19 [19,20].

Clinical data were stratified by sex, with patients analyzed separately as women and men. Additional stratification variables included age, body mass index (BMI), SARS-CoV-2 variant dominance period, and the presence of comorbidities. Age was analyzed both as a continuous variable (median with interquartile range) and as a categorical variable, with a specific focus on patients aged ≥65 years, in accordance with established thresholds associated with an increased risk of severe COVID-19 and mortality. According to the GISAID (Global Initiative on Sharing All Influenza Data) infections in Poland up to February 2021 were attributed to the pre-Alpha, those between February and June 2021 to the Alpha variant (B.1.1.7), those between July 2021 and December 2022 to the Delta variant (B.1.617.2), and those from January 2023 onwards to the Omicron variant (B.1.1.529 and sublineages). Comorbidities included obesity (defined as BMI ≥ 30 kg/m^2^), arterial hypertension, diabetes mellitus, chronic obstructive pulmonary disease (COPD), ischemic heart disease, prior myocardial infarction, stroke, malignancy, and other cardiovascular, metabolic, or respiratory diseases. Arterial hypertension was defined as a documented medical history of hypertension, current antihypertensive treatment, or repeated blood pressure measurements ≥ 140/90 mmHg during hospitalization. Diabetes mellitus was defined based on a prior diagnosis, use of antidiabetic medication, or documented hyperglycemia meeting diagnostic criteria. Malignancy was defined as any active solid or hematologic cancer or a history of cancer requiring oncological treatment within the previous five years. Baseline clinical status at hospital admission was defined based on the presence of symptoms and peripheral oxygen saturation (SpO_2_) measured on room air. Patients were classified as clinically stable or unstable with respect to respiratory status according to predefined SpO_2_ thresholds; the term “clinically unstable” refers exclusively to respiratory instability resulting from hypoxemia. Patients were categorized as follows: asymptomatic; symptomatic and clinically stable (non-hypoxemic), defined as SpO_2_ > 95%; symptomatic and clinically unstable with moderate hypoxemia (SpO_2_ 91–95%); and symptomatic and clinically unstable with severe hypoxemia (SpO_2_ ≤ 90%) or presenting with acute respiratory distress syndrome (ARDS). ARDS was defined according to standard criteria, including acute onset, bilateral pulmonary infiltrates on chest imaging, respiratory failure not fully explained by cardiac causes or fluid overload, and impaired oxygenation.

Baseline laboratory assessment included quantitative measurements of inflammatory and hematological parameters obtained at hospital admission. These comprised serum concentrations of C-reactive protein (CRP; reference range <5 mg/L), procalcitonin (PCT; <0.5 ng/mL), interleukin-6 (IL-6; <7 pg/mL), and D-dimer (<500 ng/mL); absolute counts of white blood cells (4.0–11.0 × 10^9^/L), lymphocytes (1.0–4.0 × 10^9^/L), neutrophils (2.0–7.0 × 10^9^/L), and platelets (150–400 × 10^9^/L); and serum alanine aminotransferase (ALT) activity (≤40 U/L).

Comparative analysis was performed for early clinical symptoms reported at hospital admission. The frequency of the following symptoms was systematically recorded: cough, fatigue, dyspnea, fever, disturbances of smell (anosmia), diarrhea, headache, nausea, and vomiting.

Treatment variables included the use of antiviral therapy, immunomodulatory agents, systemic corticosteroids, anticoagulation with low-molecular-weight heparin, and antibiotics administered for confirmed or suspected bacterial superinfections. All treatment decisions were made by the attending physicians in accordance with national guidelines and individual clinical assessments.

Clinical outcomes included the assessment of the percentage of patients requiring oxygen therapy or mechanical ventilation, the duration of oxygen supplementation, the length of hospitalization, and the in-hospital mortality rate expressed as a percentage. Additional endpoints comprised the age of deceased patients, the prevalence of comorbidities among non-survivors, and mortality rates for patients aged ≥65 years.

In addition, a multivariable analysis was carried out to identify predictors of mortality and the need for mechanical ventilation. The variables considered included sex, age, viral variant, comorbidities such as diabetes, stroke, and malignancy, baseline SpO_2_, oxygen therapy requirement, and selected laboratory markers including CRP, PCT, and IL-6.

Due to the retrospective design of the study, patient consent was waived. The study was performed in accordance with the principles of the Declaration of Helsinki and was approved by the Ethics Committee of the Jan Kochanowski University (16/2025).

### 2.2. Statistical Analyses

Qualitative variables are expressed as counts and percentages, while quantitative variables are presented as median and interquartile range (Q1–Q3), since none of them followed a normal distribution, which was verified using the Shapiro–Wilk test. Differences between categorical variables were assessed with the chi-squared test or Fisher’s exact test, as appropriate. Comparisons of quantitative variables were performed using the Mann–Whitney U test. A multivariable analysis was conducted using logistic regression in order to identify independent risk factors for mortality as well as independent predictors of the need for mechanical ventilation. All variables included in the models were complete, and no imputation was required. A *p*-value of <0.05 was considered statistically significant. All analyses were performed with Statistica software, version 13 (StatSoft, Tulsa, OK, USA).

## 3. Results

### 3.1. Demographic Characteristics of Patients

We analyzed data from 2811 hospitalized patients, with a slight predominance of males (50.3%) (Table 1). The median age of male patients was lower compared with females (63 vs. 67.5 years; *p* < 0.0001), with a lower proportion of men aged 65 years and older (46.7%) compared with women (57.4%) (*p* < 0.0001). BMI was comparable between the groups, although slightly higher in men (27.7 vs. 27; *p* < 0.0001). The distribution of SARS-CoV-2 variants differed significantly between women and men. During the period of Alpha variant dominance, the disease was diagnosed more often in males, whereas during the period of Omicron variant dominance, it was diagnosed more often in females. Regarding comorbidities, a higher prevalence of coexisting diseases was observed in women (79.4% vs. 74%; *p* < 0.0008) (Table 1).

**Figure 1:**
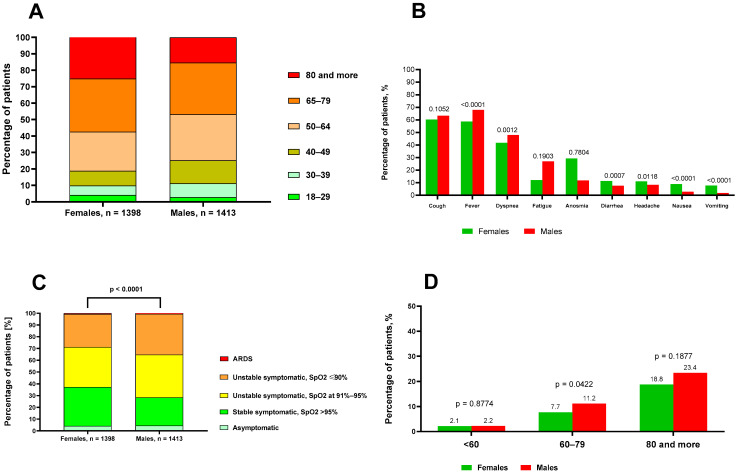
Age structure of the analyzed patients (**A**), comparison of symptom frequency between sexes (**B**), baseline clinical status at hospital admission (**C**), and mortality rate in different age groups of patients (**D**). The numbers above the bars represent *p*-values.

The age distribution of hospitalized patients differed slightly by sex. The largest group in both women and men was 65–79 (32.3% vs. 31.3%). Women were more often in the ≥80-year group (25.1% vs. 15.4%), whereas men were more often in the 50–64-year group (28.1% vs. 23.8%). Patients aged 40–49 years were also more common among men (13.9% vs. 8.9%). Younger age groups (<40 years) were infrequent in both sexes (Figure 1A).

### 3.2. Baseline Clinical Status, Symptoms, and Laboratory Parameters in the Studied Cohort

Symptom presentation differed significantly between male and female patients (Figure 1B). Fever and dyspnea were more common in men, while diarrhea, headache, nausea, and vomiting were more common in women. At admission, a higher proportion of women presented with symptomatic disease in stable condition (SpO_2_ > 95%) than men (33% vs. 24%; *p* < 0.0001). In contrast, men were more often admitted in unstable condition, with baseline SpO_2_ between 91 and 95% or ≤90%, than women (Figure 1C). Median values of WBC, lymphocytes, neutrophils, CRP, PCT, and IL-6 differed significantly across oxygen saturation groups (SpO_2_ > 95%, 91–95%, and <90%), with lower saturation associated with higher WBC, neutrophils, CRP, PCT, and IL-6, and lower lymphocyte counts (all *p* < 0.0001) (Supplementary Table S1). Among baseline laboratory parameters, significantly higher median CRP, WBC with elevated neutrophil counts, IL-6, and ALT activity were observed in male patients (*p* < 0.0001). In contrast, women showed higher lymphocyte counts and D-dimer concentrations than men (Table 2).

### 3.3. In-Hospital Treatment

In general, antiviral therapy was administered more frequently to male patients, particularly remdesivir, whereas nirmatrelvir/ritonavir was more often prescribed to female patients. Immunomodulatory treatment with tocilizumab, baricitinib, and corticosteroids (dexamethasone), given during the cytokine storm phase, was also used more frequently in men. Similarly, antibiotics for bacterial superinfections and low-molecular-weight heparin (both prophylactic and therapeutic doses) were prescribed more often to male patients (Table 3).

### 3.4. Outcomes

A significantly higher percentage of male patients required oxygen therapy compared with females (60% vs. 49.9%, *p* < 0.0001), and the need for mechanical ventilation was also more frequent among males (4.8% vs. 3.7%), but the difference did not reach statistical significance. The median duration of oxygen supplementation (8 days) and median length of hospitalization (10 days) were comparable between the sexes (Table 4). Overall mortality was higher among males than females (9.1% vs. 8.6%) (Table 4) and increased with age in both sexes. In patients younger than 60 years, mortality was very low and comparable between females and males (2.1% vs. 2.2%; *p* = 0.8774). In the 60–79 years group, mortality was significantly higher in males compared with females (11.2% vs. 7.7%; *p* = 0.0422). Among patients aged ≥80 years, mortality reached 18.8% in females and 23.4% in males; however, this difference did not reach statistical significance (*p* = 0.1877) (Figure 1C). The mortality rate was 6.2%, 8%, 12.9%, and 9.6% in the pre-Alpha, Alpha, Delta, and Omicron periods, respectively. Analysis of mortality by variant dominance and sex showed that during the pre-Alpha period, mortality was significantly higher in males than in females (*p* = 0.007), while no statistically significant sex differences were observed in the subsequent Alpha, Delta, or Omicron periods.

### 3.5. Risk Factors for Mechanical Ventilation and Death

Non-survivors were significantly older, had more comorbidities, and were more frequently infected with Omicron or Delta variants. They presented with a more severe clinical status and higher inflammatory markers, and they were more likely to require oxygen therapy or mechanical ventilation (Table 5 and Table 6). Dyspnea was significantly more common, while cough and smell/taste disturbances were less frequent.

Mortality in the overall population, as well as in female and male subgroups, increased with decreasing oxygen saturation (SpO_2_, >95%, 91–95%, <90%) across all SARS-CoV-2 variants (pre-Alpha, Alpha, Delta, and Omicron), with most differences between saturation groups being statistically significant (Supplementary Table S2). Independent predictors of mechanical ventilation included age ≥ 80 years, CRP (before treatment) > 100 mg/L, procalcitonin > 1 ng/mL, and IL-6 > 100 pg/mL (Table 7).

Multivariable analysis (Table 8) identified the following independent predictors of mortality: age ≥ 80 years, PCT > 1 ng/mL, IL-6 > 100 pg/mL, and the need for oxygen therapy at admission.

## 4. Discussion

This study provides one of the most comprehensive analyses to date of sex-based differences in the clinical course of COVID-19 among consecutively hospitalized patients at four infectious disease centers in eastern Poland through the end of 2024. Our findings confirm and extend prior observations, highlighting biological sex as a key modifier of disease progression, symptomatology, treatment patterns, and outcomes. Below, we discuss these disparities in the context of demographic characteristics, symptom presentation, laboratory and inflammatory markers, treatment approaches, and clinical outcomes, including oxygen supplementation, mechanical ventilation, and mortality, while also considering temporal changes in disease severity and variant-specific virulence across successive pandemic waves.

Our analysis revealed significant sex-related disparities in the demographic and clinical characteristics of hospitalized COVID-19 patients. Female patients were, on average, older than their male counterparts, a finding consistent with reports from diverse geographical settings [22,23]. Furthermore, a significantly greater proportion of female patients were aged ≥65 years (57.4%) compared to males (46.7%). This observed age difference among hospitalized individuals may be partially explained by sex-related differences in healthcare-seeking behavior and occupational exposure. For example, the QUALICOPC study, involving nearly 7300 patients, demonstrated that women were significantly more likely than men to report visiting their primary care providers [24]. Although the overall prevalence of obesity (BMI ≥ 30) did not differ significantly between sexes in our cohort, the median BMI was lower among female patients, which contrasts with global trends, where obesity is generally more prevalent among women than men [25]. Moreover, despite the absence of significant sex differences, obesity and diabetes have a multifactorial impact on the course of COVID-19 and worsen the overall prognosis [26]. Interestingly, our analysis revealed a higher comorbidity burden among female patients, with 79.4% having at least one comorbid condition. Specifically, conditions such as hypertension and other cardiovascular diseases were more prevalent in women, which differs from those reported in other studies. For instance, Klang et al. observed no significant sex differences in the prevalence of cardiovascular comorbidities among COVID-19 patients [23]. Similarly, an Italian study reported that 81% of hospitalized COVID-19 patients had multiple comorbidities, with no significant differences between sexes either overall or across age groups [22]. Conversely, male patients in our cohort exhibited significantly higher rates of myocardial infarction, suggesting sex-based differences in the manifestation of cardiovascular complications. This observation is consistent with a recent meta-analysis, which reported a lower risk of myocardial infarction in female patients (*p* = 0.02) [27]. Although women presented with a greater overall burden of comorbidities, the types of underlying conditions and their clinical implications appear to differ between sexes. Variant-specific analysis revealed notable sex differences in infection rates. During the Alpha variant wave, a significantly higher proportion of male patients were affected compared to females, which is opposite to reports from other studies; for example, Lonconsole et al., in a cohort of over 11,000 patients infected with the Alpha variant, reported that 52.7% of patients were female, aligning with our observations [28]. In contrast, the Omicron variant appeared to disproportionately affect female patients, a pattern that was reported differently in other countries [29].

Beyond differences in infection rates, SARS-CoV-2 variants differed substantially in their biological properties and, subsequently, clinical impact. Omicron lineages have been consistently associated with reduced intrinsic virulence compared with earlier variants such as Alpha and Delta. Experimental data indicate that Omicron is generally characterized by lower fusogenicity, a parameter that affects viral pathogenicity, as it is less dependent on TMPRSS2 and prefers the endocytic route of entry [30,31]. Moreover, and interrelated, Omicron subvariants exhibit increased tropism for the upper respiratory tract and reduced replication in lung tissue [32,33], resulting in lower rates of severe disease, pneumonia, respiratory failure, and death [5,34,35]. This reduced virulence likely contributed to the attenuation of severe outcomes observed during Omicron-dominant periods in our cohort, despite high case numbers and substantial hospital admission volumes. At the same time, Omicron and its sublineages exhibit enhanced viral fitness through increased transmissibility and marked immune-escape capacity, allowing efficient infection even in individuals with vaccine-induced or infection-acquired immunity [36,37]. This evolutionary trade-off, i.e., greater immune evasion combined with attenuated pathogenicity, has resulted in a high frequency of breakthrough infections, while overall disease severity at the population level has remained substantially lower than in earlier pandemic phases [38,39]. Available data indicate that this evolutionary trajectory has continued beyond the initial emergence of Omicron. Comparisons of clinical severity between the 2023/2024 and 2024/2025 seasons suggest a sustained trend toward milder disease courses, with further reductions in rates of severe hypoxemia, intensive care utilization, and mortality [40]. This pattern likely reflects the combined effects of ongoing viral evolution favoring immune escape rather than virulence, increasing breadth of population immunity, and improved clinical preparedness. In this setting, hospital admissions appear to be increasingly concentrated among specific and more narrow high-risk groups, such as older adults and individuals with significant comorbidities, and may more frequently reflect precautionary monitoring or the management of underlying conditions rather than severe COVID-19-driven pathology alone. Conversely, during the early pandemic phases, limited healthcare capacity and restricted access to hospital care may have resulted in left censoring, whereby the most severely ill patients were underrepresented among hospitalized cohorts, complicating direct comparisons of severity and outcomes across time [41]. Importantly, it should be stressed that vaccination continues to play a critical role in reducing the overall disease burden, even in the context of immune-evasive and less pathogenic SARS-CoV-2 (sub)variants, by preserving robust protection against severe clinical outcomes rather than infection per se [42,43,44]. Moreover, a continued surveillance of emerging SARS-CoV-2 subvariants through integrated genomic, clinical, and environmental monitoring is needed to guide public health responses and ensure that healthcare systems remain prepared for potential future shifts in transmissibility, immune escape, or disease severity [45,46].

The comparison of baseline clinical status between female and male COVID-19 patients revealed significant sex-related differences in disease severity at hospital admission. Although the proportion of asymptomatic individuals was similar, a notably higher percentage of males presented with symptoms classified as unstable, including moderate hypoxia (SpO_2_ 91–95%) and severe hypoxia (SpO_2_ ≤ 90%). Additionally, the median baseline SpO_2_ was higher in females (93%) than in males (92%) (*p* < 0.0001). These findings are consistent with previous research indicating that men tend to present with more severe symptoms already at the time of hospital admission. Several observational studies, for example, that by Jin et al. (2020), have reported that male patients are more likely to experience lower SpO_2_ levels and greater respiratory distress early in the disease course [47]. The observed sex disparities in COVID-19 severity may be attributed to a combination of biological and behavioral factors. Biologically, women often mount a stronger immune response, potentially due to estrogen’s modulatory effects and two X chromosomes, which contain several immune-related genes. Men, on the other hand, may have a less effective antiviral immune response, possibly influenced by testosterone and a higher prevalence of risk factors such as hypertension, cardiovascular disease, and smoking [48].

Our data show notable sex-based differences in symptom presentation. Males more frequently reported fever and dyspnea, while females had higher rates of gastrointestinal symptoms such as nausea, vomiting, and diarrhea. However, it is important to note that COVID-19 symptomatology has evolved significantly over time and varies by viral variant. For example, anosmia was common early in the pandemic but became less frequent with later variants like Omicron [3]. The predominance of upper respiratory and systemic symptoms during Omicron waves, together with reduced pulmonary involvement, may partly explain the lower need for advanced respiratory support and the overall decline in disease severity observed in later periods.

Antiviral treatment was mostly remdesivir-based and was administered significantly more often in men than in women (35.2% vs. 30%, *p* = 0.0003). Despite females being older and more burdened by comorbidities, men presented in a worse clinical state at the time of admission, with lower saturation and higher inflammatory markers, indicating a more robust immunological reaction. These results align with previous studies conducted on the Polish population in different waves of the pandemic [3,4]. The lower SpO_2_ is reflected in a higher percentage of male patients requiring oxygen supplementation (60% vs. 49.9%, *p* < 0.0001). A retrospective cohort study based in the USA showed that males were 32% more likely to present with hypoxemia compared to female patients [49]. Overall, males are considered more susceptible to respiratory tract infections and are characterized by a more severe disease course [50]. Several factors contribute to this phenomenon, such as smoking addiction, which is more common among men, as more than one/third of men in the world smoke tobacco compared to less than one in ten women [51]. Not without influence is also the fact that women are often more exposed to respiratory tract infections, such as COVID-19, and develop stronger infection-acquired immunity. According to Eurostat, in 2020 [52], women constituted 78% of all health workers in the EU. Perception of the disease and the readiness to seek medical attention also play a role, as females generally engage with healthcare more readily than males, therefore presenting earlier and in a better clinical state at admission [53].

The androgens’ influence explains the observed constellation of younger age, fewer comorbidities, but worse clinical state at admission, with a higher inflammatory response in men. To enter the host cell, the spike protein of SARS-CoV-2 has to interact with the transmembrane protease serine 2 receptor and bind with the angiotensin-converting enzyme 2 receptor. By binding with the androgen receptor, androgens can increase the expression of transmembrane protease, serine 2, facilitating the entry of the virus into the host cells, promoting a more severe course of the disease [54]. The sex hormones’ influence requires further study, as small Italian and German analyses indicate that low testosterone promotes a more severe disease course [55,56].

The second most often used antiviral was orally administered molnupiravir, followed closely by nilmatrevir/ritonavir. Paxlovid was used significantly more often in females than in males (2.1% vs. 1.1%, *p* = 0.02). The most frequent morbidities, such as heart arrhythmias requiring anticoagulant treatment (atrial fibrillation) and hyperlipidemia, are more prevalent in males, and the drugs administered for treatment of those conditions are usually contraindicated for use with Paxlovid [57,58,59].

Immunomodulators were administered significantly more often in males than in females, which aligns with a more advanced disease stage and higher baseline inflammatory parameters, such as IL-6, in males. Dexamethasone, the uptake of which increased during the pandemic following the publication of the RECOVERY trial [60], was used significantly less frequently among women than among men (33.9% vs. 42.5%, *p* < 0.0001), reflecting a more stable clinical course of the disease, a better response to antiviral treatment, and higher effectiveness of tocilizumab. Tocilizumab initiation was more common among men than among women (11.1% vs. 6.4%), indicating that males more often entered the third stage of COVID-19, known as cytokine storm, reflected by significantly higher mediants of IL-6 levels (34 vs. 23, *p* < 0.0001). Our findings are supported by a previous meta-analysis, in which the circulating levels of IL-6 were significantly elevated in males. It was also found to be higher among those with a more severe course of the disease and those who died [61]. Anticoagulant therapy with low-molecular-weight heparin (LMWH) in prophylactic doses was initiated in most patients, regardless of sex (68.8% females vs. 72.4% males), in accordance with recommendations from the Polish Society of Epidemiologists and Infectious Disease Physicians. It was introduced as a supportive treatment in patients with risk factors for deep vein thrombosis/pulmonary embolism assessed at admission. Thromboprophylaxis, especially in older patients infected with SARS-CoV-2, is safe and effective, even in those with a high bleeding risk [62].

In our study, mortality was slightly higher in percentage terms in men than in women (9.1% vs. 8.6%), but statistically comparable between the groups. However, analysis of mortality by variant dominance revealed that during the pre-Alpha period, mortality was significantly higher in males compared with females (*p* = 0.007). Moreover, in the multiple logistic regression, IL-6 levels over 100 pg/mL, elevated PCT, age over 80 years, and the need for oxygen therapy were found to be independent factors increasing the risk of death for the whole population. Additional age-based analysis showed that in the 60–79 age group, men had significantly higher mortality than women (*p* = 0.04). In the ≥80 and <60 years brackets, men died more often than females, but there was no significant difference (23.4% vs. 18.8% and 2.2% vs. 2.1%, respectively). A study investigating in-hospital mortality in Poland found that the odds ratio for in-hospital death increased with age, and male sex and emergency admission were factors associated with higher mortality in nonsurgical departments, which supports our findings [63]. Another analysis targeting the SARS-CoV-2-infected population of over ten million patients reported that men not only had a 22% higher risk of severe COVID-19 than women, but they were also at a higher risk for intensive care unit admittance and death [64]. Although both death and mechanical ventilation were observed more often in the male population, the difference was significant only for the 60–79 years bracket.

Our stratified analysis of patients according to SpO_2_ levels (>95%, 91–95%, and <90%) demonstrated that lower oxygen saturation was consistently associated with higher mortality across all SARS-CoV-2 variants. Even in the context of reduced Omicron virulence, low oxygen saturation at admission retained prognostic value, emphasizing the continued importance of early detection and monitoring. These results highlight the potential clinical importance of early detection of SpO_2_ < 95%, as the timely recognition of hypoxemia may allow for earlier interventions and improved patient outcomes. Furthermore, our findings suggest that certain patient profiles, particularly those with multiple or high-risk comorbidities, may benefit most from prehospital or early hospital monitoring of oxygen saturation. Implementing such monitoring strategies could help prioritize care and optimize resource allocation during pandemic surges.

Men who died were significantly younger than deceased women (76 years vs. 80.5 years, *p* = 0.016, respectively). The results align with the overall trend present in the Polish population. According to the report of the Central Statistics Office published in 2023, men have higher mortality rates than women, and nearly 44% of men do not reach the age of 75 years [65]. In this instance, the younger age at the time of death in men may be explained by the overall younger age at admission, combined with a worse clinical state at baseline and higher inflammatory markers compared to women. Men were also more prone to secondary bacterial infections and venous thromboembolic events, seen in the greater use of antibiotics and therapeutic doses of low-molecular-weight heparin. Both are associated with a worse prognosis and are considered more common among patients with higher BMI (27.7 kg/m^2^ vs. 27 kg/m^2^, *p* < 0.0001, in males and females, respectively) [66,67,68,69]. The decision to initiate antibiotic treatment was based on several factors, including the appearance of consolidation on chest computer tomography, worsening of the clinical state, and an increase in inflammatory laboratory parameters, consistent with the prescription rationale used in other studies [70]. Additionally, the internal hospital recommendations for antibiotic use were implemented. However, antibiotics can also be prescribed in the absence of the aforementioned factors due to uncertainty, in patients in a severe clinical state with multimorbidity, especially in outpatient settings [70]. Overtreatment with antibiotics leads to the development of antibiotic resistance in bacteria and narrows the possibilities of therapy in the future [71]. A Polish study assessing antibiotic consumption underscores the urgent need for improved implementation of antimicrobial stewardship policies [72]. It should be combined with on-site infectious disease specialist consultations and more frequent shortening of antibiotic therapy [71]. Moreover, it is worth noting that PCT, a peptide whose extra-thyroid synthesis increases in the event of bacterial infection, was notably higher in men than women, and was ruled an independent factor increasing the risk of death and mechanical ventilation [73]. The time of hospitalization was also longer for men, and this is important information as the hospital stay on its own carries a 17.6% risk of infection that increases each day by 1.6% [74].

This study has several limitations that should be acknowledged. First, our analysis did not include vaccination status as a variable. Although vaccination is a critical factor influencing disease severity and outcomes, numerous confounding elements—such as the number of vaccine doses received, time elapsed since the last dose, type of vaccine administered, and history of prior SARS-CoV-2 infection—make it challenging to draw meaningful conclusions using simple stratification (e.g., vaccinated vs. unvaccinated). Moreover, accurate data regarding these variables were inconsistently available across all study sites. Second, SARS-CoV-2 variant identification was not based on individual genomic sequencing but inferred from epidemiological surveillance data, indicating periods of variant predominance in Poland. This indirect approach may have led to misclassification, especially during transitional periods between dominant strains. Another limitation concerns the heterogeneity of reasons for hospital admission. In particular, during the Omicron wave, a proportion of patients may have been admitted for non-COVID-related conditions and incidentally tested positive for SARS-CoV-2. Differentiating between admissions due to COVID-19 versus incidental infection is inherently challenging in retrospective analyses and may have introduced bias in assessing disease severity and outcomes. Additionally, as an observational study based on real-world evidence and retrospective data, our analysis is inherently susceptible to several forms of bias. These include potential inaccuracies in electronic medical records, such as data entry errors or missing values, and underreporting of less prominent clinical features or adverse events. Despite these limitations, the study has notable strengths. Including data from four geographically distinct centers enhances the generalizability of our findings. By capturing a real-world population over an extended time frame—including multiple waves and variants—we provide a robust and comprehensive view of sex-specific differences in COVID-19 presentation and outcomes.

Despite advances in understanding COVID-19, several questions remain. Future research should focus on integrating vaccination status, prior infection history, and more precise genomic characterization of SARS-CoV-2 variants to refine risk stratification models. Although vaccination data were unavailable at the individual level, national vaccination coverage in Poland varied substantially across the analyzed periods. The vaccine uptake increased markedly during 2021, coinciding with the Alpha and Delta variant waves, and stabilized thereafter. However, these national trends cannot be directly compared with mortality observed among hospitalized patients in our study, as hospital admission patterns, clinical profiles, and timing of infection likely differed from those of the general population. Nonetheless, it is plausible that increasing population-level immunity, resulting from both vaccination and prior infection, contributed to the overall decline in COVID-19 severity as reported in other Polish studies [5,6,40]. This contextual information should be considered when interpreting temporal differences in in-hospital mortality across variant periods. Prospective studies could examine the interplay between sex, age, comorbidities, and immune response in greater detail to elucidate the mechanisms underlying differential disease severity. Additionally, there is a need for developing predictive scoring systems that combine clinical features and laboratory markers such as PCT and IL-6 to facilitate the early identification of high-risk patients. Long-term follow-up studies may also provide insight into the relationship between acute disease severity, systemic inflammation, and post-COVID sequelae, helping guide post-hospital care and rehabilitation strategies. Ultimately, continued research in these areas can enhance personalized management of COVID-19 and strengthen preparedness for future infectious disease challenges.

## 5. Conclusions

This multicenter retrospective study of 2811 hospitalized COVID-19 patients in eastern Poland demonstrates that sex influences the clinical course, but not independently the mortality risk, once age and inflammatory burden are accounted for. Female patients were generally older and had more comorbidities, whereas male patients presented with more severe respiratory compromise, higher systemic inflammation, and received more intensive pharmacological treatment. Independent predictors of mortality and mechanical ventilation were elevated PCT, high IL-6 concentrations, and oxygen requirement at admission, while advanced age further amplified the risk. These results indicate that outcome disparities are better explained by biological age and host inflammatory response than by sex alone. Taken together, our findings emphasize the need for early recognition of systemic inflammation and respiratory compromise as key prognostic indicators. This knowledge may support timely triage, targeted therapeutic decisions, and more precise risk stratification in hospitalized COVID-19 patients, particularly in resource-limited settings where rapid identification of high-risk individuals remains crucial. Recognizing sex-related differences in disease severity and inflammatory profiles can help clinicians tailor management strategies and optimize hospital resource allocation. These findings may improve patient outcomes, particularly in settings with limited resources, by enabling rapid identification of high-risk individuals and informed clinical decision-making. Further surveillance of the clinical picture of COVID-19 is advocated as SARS-CoV-2 continues to evolve, and the exact outcomes of these emerging (sub)lineages on disease severity, treatment needs, and mortality remain uncertain.

## Figures and Tables

**Table 1:** The demographic and clinical characteristics of the analyzed patients (*n* = 2811).

Parameters	Females, *n* = 1398	Males, *n* = 1413	*p*-Value
Age (years), median (Q1, Q3) (min–max)	67.5 (55–80)	63 (49–74)	<0.0001
Age ≥ 65 years, *n* (%)	802 (57.4)	660 (46.7)	<0.0001
BMI (kg/m^2^), median (Q1, Q3)	27 (23.4–31.1)	27.7 (24.7–31)	<0.0001
Variants dominance periods, *n* (%)			
pre-Alpha	402 (28.7)	418 (29.6)	0.6295
Alpha	308 (22)	388 (27.5)	0.0009
Delta	275 (19.8)	280 (19.8)	0.923
Omicron	413 (29.5)	327 (23.1)	0.0001
Comorbidities, *n* (%)			
Any Comorbidity	1110 (79.4)	1046 (74)	0.0008
Obesity (BMI ≥ 30)	404 (28.9)	425 (30.1)	0.47
Hypertension	775 (55.4)	696 (49.3)	0.001
Diabetes	290 (20.7)	280 (19.8)	0.5407
Stroke	71 (5.1)	85 (6)	0.278
COPD	49 (3.5)	70 (5)	0.0564
MI	51 (3.6)	86 (6.1)	0.0027
Malignant tumor	123 (8.8)	108 (7.6)	0.2649
Ischemic heart disease	199 (14.2)	204 (14.4)	0.8781
Other CVD	310 (22.2)	251 (17.8)	0.0034
Other respiratory diseases	102 (7.3)	103 (7.3)	0.9946
Other metabolic diseases	96 (6.9)	98 (6.9)	0.9428
Others	735 (52.6)	608 (43)	<0.0001
Comedications	1110 (79.4)	1052 (74.5)	0.0018
Saturation at the baseline, median (Q1, Q3)	93 (90–96)	92 (89–95)	<0.0001

Abbreviations: BMI, body mass index; COPD, chronic obstructive pulmonary disease; COVID-19, coronavirus disease 2019; CVD, cardiovascular disease; MI, myocardial infarction.

**Table 2:** Laboratory parameters of analyzed patients (*n* = 2811).

Parameters [Median (Q1–Q3)]	Females, *n* = 1398	Males, *n* = 1413	*p*-Value
CRP, mg/L	32 (10–73.9)	56.3 (21–113.1)	<0.0001
PCT, ng/mL	0.1 (0.1–0.1)	0.1 (0.1–0.2)	<0.0001
WBC, ×10^3^/µL	5420 (4045–7310)	5930 (4560–8040)	<0.0001
Lymphocytes, ×10^3^/µL	1100 (790–1600)	1060 (750–1500)	0.051
Neutrophils, ×10^3^/µL	3560 (2330–5260)	4010 (2770–6000)	<0.0001
NLR	3.1 (1.9–5.8)	3.8 (2.2–6.8)	<0.0001
Platelets, ×10^3^/µL	195,500 (153,000–249,500)	177,000 (138,000–232,000)	<0.0001
IL-6, pg/mL	23 (9.5–53)	34 (13–77.8)	<0.0001
d-dimer, ng/mL	827 (506.2–1455)	790 (471–1337)	0.0248
ALT, IU/L	25 (17–40)	32.5 (22–55)	<0.0001

Abbreviations: ALT, alanine aminotransferase; CRP, C-reactive protein; IL-6, interleukin-6; NLR, neutrophil-to-lymphocyte ratio; PCT, procalcitonin; WBC, white blood cell.

**Table 3:** Pharmacological treatment by gender in analyzed patients (*n* = 2811).

Parameters	Females, *n* = 1398	Males, *n* = 1413	*p*-Value
Antivirals, *n* (%)			
Remdesivir	420 (30)	498 (35.2)	0.0003
Molnupiravir	40 (2.9)	32 (2.3)	0.3168
Casirivimab/Imdevimab	3 (0.2)	3 (0.2)	>0.9999
Nirmatrelvir/Ritonavir	30 (2.1)	15 (1.1)	0.022
Immunomodulators, *n* (%)			
Tocilizumab	90 (6.4)	157 (11.1)	<0.0001
Dexamethason	474 (33.9)	615 (43.5)	<0.0001
Baricitinib	3 (0.2)	4 (0.3)	>0.9999
Antibiotics, *n* (%)	12 (0.9)	17 (1.2)	0.3658
Low-molecular-weight heparin, *n* (%)			
prophylactic dose	962 (68.8)	1023 (72.4)	0.0369
therapeutic dose	109 (7.8)	117 (8.3)	0.6374

**Table 4:** Outcomes in analyzed patients (*n* = 2811).

Parameters	Females, *n* = 1398	Males, *n* = 1413	*p*-Value
Need for oxygen supplementation, *n* (%)	698 (49.9)	848 (60)	<0.0001
Need for mechanical ventilation, *n* (%)	52 (3.7)	68 (4.8)	0.1518
Time of oxygen supplementation (days), median (Q1, Q3)	8 (5–12)	8 (5–12)	0.3529
Time of hospitalization (days), median (Q1, Q3)	10 (7-13)	10 (8–14)	0.0105
Mortality, *n* (%)	120 (8.6)	129 (9.1)	0.6106
Age of patients who died (years), median (Q1, Q3) (min–max)	80.5 (70.5–87.5) (30–99)	76 (67–85) (25–98)	0.0155
Comorbidities of patients who died, *n* (%)	112 (93.3)	118 (91.5)	0.5806
Mortality in patients with age ≥65 years, *n* (%)	105 (13.1)	107 (16.2)	0.0918
Mortality by variants dominance periods, *n* (%)	*N* = 120	*N* = 129	
pre-Alpha	16 (13.3)	35 (27.1)	0.007
Alpha	32 (26.7)	24 (18.6)	0.1279
Delta	38 (31.7)	33 (25.6)	0.2879
Omicron	34 (28.3)	37 (28.7)	0.9514

**Table 5:** Comparison of baseline characteristics, comorbidities, clinical status, and laboratory parameters between surviving and deceased patients.

Parameters	No Deaths, *n* = 2562	Deaths, *n* = 249	*p*-Value
Gender, male/female, *n* (%)/*n* (%)	1284 (50.1)/1278 (49.9)	129 (51.8)/120 (48.2)	0.6106
Age (years), median (Q1–Q3) (min–max)	64 (50–75) (18–98)	78 (68–86) (25–99)	<0.0001
Age ≥ 65 years, *n* (%)	1250 (48.8)	212 (85.1)	<0.0001
BMI (kg/m^2^), median (Q1–Q3)	27.4 (24.2–31)	26 (23.2–31.1)	0.0036
Variants dominance periods, *n* (%)			0.0003
pre-Alpha	769 (30)	51 (20.5)	
Alpha	640 (25)	56 (22.5)	
Delta	484 (18.9)	71 (28.5)	
Omicron	669 (26.1)	71 (28.5)	
Comorbidities, *n* (%)			
Any Comorbidity	1926 (75.2)	230 (92.4)	<0.0001
Obesity (BMI ≥ 30 kg/m^2^)	763 (29.8)	66 (26.5)	0.7308
Hypertension	1301 (50.8)	170 (68.3)	<0.0001
Diabetes	496 (19.4)	175 (70.3)	0.0001
Stroke	131 (5.1)	25 (10)	0.0012
COPD	99 (3.9)	20 (8)	0.0018
Myocardial Infarction	118 (4.6)	19 (7.6)	0.0343
Malignant tumor	191 (7.5)	40 (16.1)	<0.0001
Ischemic heart disease	341 (13.3)	187 (75.1)	<0.0001
Other CVD	480 (18.7)	81 (32.5)	<0.0001
Other respiratory diseases	179 (7)	26 (10.4)	0.0453
Other metabolic diseases	179 (7)	15 (6)	0.5672
Others	1182 (46.1)	161 (64.7)	<0.0001
Comedications, *n* (%)	1947 (76)	215 (86.3)	<0.0001
Baseline clinical status on hospital admission, *n* (%)			<0.0001
Asymptomatic	113 (4.4)	9 (3.6)	
Stable symptomatic, SpO_2_ > 95%	784 (30.6)	17 (6.8)	
Unstable symptomatic, SpO_2_ at 91–95%	944 (36.8)	42 (16.9)	
Unstable symptomatic, SpO_2_ ≤ 90%	717 (28)	163 (65.5)	
ARDS	4 (0.2)	18 (7.2)	
Symptoms, *n* (%)			
Cough	1612 (62.9)	125 (50.2)	<0.0001
Fever	1633 (63.7)	146 (58.6)	0.1106
Dyspnea	1099 (42.9)	163 (65.5)	<0.0001
Disturbances of smell and taste	324 (12.6)	9 (3.6)	<0.0001
Diarrhea	240 (9.4)	25 (10)	0.7288
Headache	258 (10.1)	14 (5.6)	0.0234
Nausea	156 (6.1)	8 (3.2)	0.0645
Vomiting	123 (4.8)	11 (4.4)	0.7864
Fatigue	726 (28.3)	65 (26.1)	0.4544
Saturation at the baseline, median (Q1, Q3)	93 (90–96)	86 (80–90)	<0.0001
Lung abnormalities in CT, *n* (%)	1703 (66.5)	177 (71.1)	0.001
The need for oxygen therapy, *n* (%)	1316 (51.4)	230 (92.4)	<0.0001
The need for mechanical ventilation, *n* (%)	24 (0.9)	96 (38.6)	<0.0001

Abbreviations: ARDS, acute respiratory distress syndrome; BMI, body mass index; COPD, chronic obstructive pulmonary disease; CT, computed tomography; CVD, cardiovascular disease; Q1–Q3, first to third quartile; SpO_2_, peripheral capillary oxygen saturation.

**Table 6:** Comparison of laboratory parameters between surviving and deceased patients.

Parameters	No Deaths, *n* = 2562	Deaths, *n* = 249	*p*-Value
CRP, mg/L, median (Q1–Q3)	39.7 (12.9–85.7)	101 (53.3–182.5)	<0.0001
CRP > 100 mg/L, *n (%)*	544 (21.2)	124 (49.8)	<0.0001
PCT, ng/mL, median (Q1–Q3)	0.1 (0.1–0.1)	0.3 (0.1–1)	<0.0001
PCT > 1 ng/mL, *n (*%)	93 (3.6)	61 (24.5)	<0.0001
WBC, ×10^3^/µL, median (Q1–Q3)	5610 (4240–7550)	6750 (4950–10110)	<0.0001
Lymphocytes, ×10^3^/µL, median (Q1–Q3)	1100 (795–1570)	880 (540–1200)	<0.0001
Neutrophils, ×10^3^/µL, median (Q1–Q3)	3700 (2480–5480)	4910 (3490–8110)	<0.0001
NLR, median (Q1–Q3)	3.3 (1.9–5.9)	6.2 (3.4–10.8)	<0.0001
Platelets, ×10^3^/µL, median (Q1–Q3)	189 (147–242)	165 (120–212)	<0.0001
IL-6, pg/mL, median (Q1–Q3)	25.8 (10.3–56.3)	83 (43.2–162.3)	<0.0001
IL-6 > 100 pg/mL, *n* (%)	288 (11.2)	95 (38.2)	<0.0001
d-dimer, ng/mL, median (Q1–Q3)	770 (471–1303)	1436.5 (867–2961.9)	<0.0001
ALT, IU/L, median (Q1–Q3)	28 (19–46)	32 (19–55)	0.031
Lung abnormalities in CT, *n* (%)	1703 (66.5)	177 (71.1)	0.001

Abbreviations: ALT, alanine aminotransferase; CRP, C-reactive protein; IL-6, interleukin-6; NLR, neutrophil-to-lymphocyte ratio; PCT, procalcitonin; Q1–Q3, first to third quartile; WBC, white blood cell.

**Table 7:** Multivariable analysis of demographic and laboratory predictors of mechanical ventilation in COVID-19 patients.

Effect	OR	95% Cl	Effect Measure	Wald Stat	*p*-Value
Gender	0.76	0.5–1.15	Male	1.65	0.1989
Age	7.14	1.75–33.33	80 and more	7.53	0.0061
SARS-CoV-2 variant	1.22	0.75–1.98	Delta	0.65	0.4211
Comorbidities	0.76	0.48–1.20	Yes	1.40	0.2372
DM	0.79	0.40–1.55	Yes	0.47	0.4928
Stroke	1.11	0.34–3.68	Yes	0.03	0.8635
SpO_2_ at baseline (without oxygen therapy)	0.85	0.53–1.35	≥90	0.47	0.4926
CRP (before treatment)	1.82	1.15–2.88	>100 mg/L	6.56	0.0104
Procalcitonin (before treatment)	2.09	1.20–3.63	>1 ng/mL	6.74	0.0094
IL-6 (before treatment)	2.30	1.40–3.78	>100 pg/mL	10.88	0.0010

Abbreviations: CI, confidence interval; CRP, C-reactive protein; DM, diabetes mellitus; IL-6, interleukin-6; OR, odds ratio; SpO_2_, peripheral capillary oxygen saturation. All variables included in the models were complete, and no imputation was required.

**Table 8:** Multivariable analysis of demographic and laboratory predictors of death in COVID-19 patients.

Effect	OR	95% Cl	Effect Measure	Wald Stat	*p*-Value
Gender	1.11	0.80–1.55	Male	0.38	0.948
Age	3.78	2.66–5.39	80 and more	54.31	0.003
BMI	1.19	0.82–1.72	30 and more	0.86	0.627
SARS-CoV-2 variant	1.48	1.02–2.15	Delta	4.30	0.62
Comorbidities	2.53	1.33–4.80	Yes	8.02	0.113
DM	1.29	0.90–1.85	Yes	1.90	0.524
Malignant	1.74	1.07–2.82	Yes	5.05	0.747
CRP	1.35	0.93–1.95	>100 mg/L	2.56	0.335
Procalcitonin	4.07	2.54–6.52	>1 ng/mL	34.08	<0.001
IL-6	2.24	1.53–3.27	>100 pg/mL	17.31	0.001
The need for oxygen therapy	9.41	5.22–16.97	Yes	55.53	<0.001

Abbreviations: BMI, body mass index; CI, confidence interval; CRP, C-reactive protein; DM, diabetes mellitus; IL-6, interleukin-6; OR, odds ratio. All variables included in the models were complete, and no imputation was required.

## Data Availability

The data supporting the reported results can be provided upon request to the corresponding author.

## Supplementary Materials

The following supporting information can be downloaded at: https://www.mdpi.com/article/10.3390/jcm15031207/s1, Table S1: Stratified analysis of, laboratory parameters according to SpO_2_ at hospital admission.; Table S2: Stratified analysis of SARS-CoV-2 variants, laboratory parameters, and mortality according to SpO_2_ at hospital admission.

## Abbreviations

The following abbreviations are used in this manuscript:

ACE2	Angiotensin-Converting Enzyme 2
AF	Atrial Fibrillation
ALT	Alanine Aminotransferase
ARDS	Acute Respiratory Distress Syndrome
BMI	Body Mass Index
COPD	Chronic Obstructive Pulmonary Disease
COVID-19	Coronavirus Disease 2019
CRP	C-Reactive Protein
CT	Computed Tomography
DNA	Deoxyribonucleic Acid
EU	European Union
GISAID	Global Initiative on Sharing All Influenza Data
ICU	Intensive Care Unit
IL-6	Interleukin-6
LMWH	Low-Molecular-Weight Heparin
mRNA	Messenger Ribonucleic Acid
NLR	Neutrophil-to-Lymphocyte Ratio
PCT	Procalcitonin
PCR	Polymerase Chain Reaction
RNA	Ribonucleic Acid
SARS-CoV-2	Severe Acute Respiratory Syndrome Coronavirus 2
SpO_2_	Peripheral Oxygen Saturation
TMPRSS2	Transmembrane Serine Protease 2
VOI	Variant of Interest
VOC	Variant of Concern
VUM	Variant Under Monitoring
WHO	World Health Organization
