# Supplementary material for: Predictors of Severe Outcomes in COVID-19: Evidence from Real-World Multicenter Retrospective Study (2020–2024)
Source: J Clin Med. 2026 Feb 3;15(3):1207. doi: 10.3390/jcm15031207 (PMC12898750; doi:10.3390/jcm15031207)
Supplement: Supplementary file 1 [file jcm-15-01207-s001.zip › jcm-4085144-supplementary.pdf]

Supplementary Table S1. Stratified analysis of, laboratory parameters according to SpO_2 at hospital admission.

**Parameters**	**Sp >95%, n=779**	**Sp 91-95%, n=1089**	**Sp <90%, n=943**	p
WBC, x 10^3/μl, median (Q1-Q3)	5280 (4120-6900)	5555 (4155-7475)	6290 (4680-8600)	<0.0001[2,3]
Lymphocytes, x 10^3/μl, median (Q1-Q3)	1375 (1000-1840)	1080 (800-1490)	890 (610-1270)	<0.0001 [1,2,3]
Neutrophils, x 10^3/μl, median (Q1-Q3)	3200 (2130-4540)	3700 (2540-5400)	4700 (3200-6805)	<0.0001 [1,2,3]
CRP, mg/L, median (Q1-Q3)	14.8 (3.7-42.2)	41 (15.9-79.8)	80 (40.5-151.8)	<0.0001 [1,2,3]
PCT, ng/mL, median (Q1-Q3)	0.1 (0.04-0.1)	0.1 (0.05-0.2)	0.1 (0.06-0.3)	<0.0001 [1,2,3]
IL-6, pg/mL, median (Q1-Q3)	12.5 (4.5-27.6)	29.1 (12.5-59)	53.4 (23.6-104)	<0.0001 [1,2,3]

Abbreviations: WBC, White Blood Cells; Sp, Oxygen saturation; CRP, C-reactive protein; PCT, Procalcitonin; IL-6, Interleukin 6; Q1-Q3, First and third quartile; n, Number of patients.

[1] Post-hoc test showed signifficant difference for Sp >95% vs Sp 91-95%

[2] Post-hoc test showed signifficant difference for Sp >95% vs Sp <90%

[3] Post-hoc test showed signifficant difference for Sp 91-95% vs Sp <90%

Supplementary Table S2. Stratified analysis of SARS-CoV-2 variants, laboratory parameters, and mortality according to SpO_2 at hospital admission.

**Parameters**	**Sp >95%, n=779**	**Sp 91-95%, n=1089**	**Sp <90%, n=943**	**P for Sp >95% vs Sp 91-95%**	**P for Sp >95% vs Sp <90%**	**P for Sp 91-95% vs Sp <90%**
General population, n (%)	16 (2.1)	46 (4.2)	187 (19.8)	0.0098	<0.0001	<0.0001
pre-Alpha	6 (0.8)	9 (0.8)	36 (3.8)	0.8932	<0.0001	<0.0001
Alfa	2 (0.3)	8 (0.7)	46 (4.9)	0.2087	<0.0001	<0.0001
Delta	4 (0.5)	10 (0.9)	57 (6.0)	0.4188	<0.0001	<0.0001
Omicron	4 (0.5)	19 (1.7)	48 (5.1)	0.0185	<0.0001	<0.0001
Females, n (%)	6 (0.8)	19 (1.7)	95 (10.1)	0.0707	<0.0001	<0.0001
pre-Alpha	2 (0.3)	2 (0.2)	12 (1.3)	>0.9999	0.0276	0.0049
Alfa	0 (0.0)	3 (0.3)	29 (3.1)	0.2703	<0.0001	<0.0001
Delta	3 (0.4)	5 (0.5)	30 (3.2)	>0.9999	<0.0001	<0.0001
Omicron	1 (0.1)	9 (0.8)	24 (2.5)	0.0527	<0.0001	0.0022
Males, n (%)	10 (1.3)	27 (2.5)	92 (9.8)	0.0674	<0.0001	<0.0001
pre-Alpha	4 (0.5)	7 (0.6)	24 (2.5)	0.7706	0.0008	0.0005
Alfa	2 (0.3)	5 (0.5)	17 (1.8)	0.7062	0.0019	0.0035
Delta	1 (0.1)	5 (0.5)	27 (2.9)	0.4105	<0.0001	<0.0001
Omicron	3 (0.4)	10 (0.9)	24 (2.5)	0.2592	0.0003	0.0043
